# A preliminary nontargeted lipidomics analysis reveals greater acylcarnitine in dark-cutting beef longissimus lumborum across visual severity levels

**DOI:** 10.1093/jas/skaf460

**Published:** 2026-01-06

**Authors:** Keayla M Harr, Madelyn A Scott, Eduardo S. P. Santos, Nara R B Cônsolo, Logan Johnson, Gretchen G Mafi, Morgan M Pfeiffer, Ranjith Ramanathan

**Affiliations:** Department of Animal and Food Science, Oklahoma State University, Stillwater, OK 74075; Department of Animal and Food Science, Oklahoma State University, Stillwater, OK 74075; College of Animal Science and Food Engineering, University of São Paulo, Pirassununga, Brazil; School of Veterinary Medicine and Animal Science, University of São Paulo, Pirassununga, Brazil; Department of Animal and Food Science, Oklahoma State University, Stillwater, OK 74075; Department of Animal and Food Science, Oklahoma State University, Stillwater, OK 74075; Department of Animal and Food Science, Oklahoma State University, Stillwater, OK 74075; Department of Animal and Food Science, Oklahoma State University, Stillwater, OK 74075

**Keywords:** acylcarnitine, beef, dark-cutting, high-pH, lipidomics, omics

## Abstract

Dark-cutting beef continues to remain one of the challenges for the global beef industry. The objective of this study was to determine the impact of varying visual degrees of dark-cutting condition on the lipidome profiles of beef *longissimus lumborum*. Beef carcasses (*n *= 6/treatment; 24 total loins) were identified at the time of grading based on the visual severity of dark-cutting levels, and beef strip loins (*longissimus lumborum*) were collected from these carcasses following fabrication. Treatments included a normal, bright cherry-red control (pH = 5.54), shady dark-cutting (half dark; pH = 5.96), moderate dark-cutting (two-thirds dark; pH = 6.38), and moderately severe dark-cutting (full dark; pH = 6.55). Approximately after 48 to 60 h postmortem, two steaks were sliced off the anterior end of each loin. The first steak was used for bloom color analysis and the second steak was used for nontargeted lipidomoc analysis using a a liquid chromatography massspectrometry approach. A total of 379 lipids, representing different classes of lipids, were identified across the 4 treatments. Pairwise comparisons revealed 21, 22, and 23 lipid species that differed (*P *< 0.05) in shady, moderate, and moderately severe dark-cutting beef, respectively, compared with normal beef. Only one lipid species (acylcarnitine 22:2) differed between moderately severe and moderate groups. Acylcarnitine species of varying carbons and saturations were the most common of the shared species in dark-cutting samples. Medium- and long-chain acylcarnitine levels were significantly different (*P *< 0.05) between dark-cutting treatments and normal pH. The importance projection analysis indicated that acylcarnitine 20:2, 18:1, and 16:0 were the top 3 lipid species contributing to differences between dark-cutting severities and normal steaks. The relative proportion of lipids involved in energy metabolism was greater (*P *< 0.05) in moderate and moderately severe than in normal bright red steaks. Greater acylcarnitine levels in postmortem muscle suggest mobilization of fatty acids for energy homeostasis in dark-cutting beef and altered metabolism.

## Teaser

Dark-cutting beef has more acylcarnitine than normal-pH beef. The current study offers further evidence that changes in energy metabolism result in high postmortem muscle pH and dark-cutting beef.

## Introduction

Dark-red to nearly coffee-bean shade of dark-cutting beef indicates a color deviation from the typical bright cherry-red. The incidence of dark-cutting beef in the United States is about 1.8% (Mayer et al., 2024), while studies in Mexico have reported rates up to 13.5% (Loredo-Osti et al., 2019). Dark appearance leads to decreased consumer acceptance (English et al., 2016; Leighton et al., 2023) and significant economic losses (Harr et al., 2024). Current knowledge suggests that chronic stress before slaughter results in depleted muscle glycogen, leading to a greater-than-normal muscle pH (Kiyimba et al., 2024). In support, enzymes and metabolites involved in glycogen metabolism were lower in dark-cutting beef, leading to muscle compensatory changes to maintain energy regulation. Although lipids constitute a significant energy source when carbohydrate levels in the tissue are low, there is limited knowledge about changes in lipid profiles in dark-cutting beef.

Cattle undergoing both acute and chronic stress are likely to deplete all available immediate energy stores, leading to the utilization of alternative energy sources to maintain cellular energy balance. More specifically, during stress, glycogen, phosphocreatine, and glucose are rapidly mobilized to sustain homeostasis and ATP levels within muscle tissue (Matarneh et al., 2021, 2023). Further, fat and glycogen stores are mobilized from liver (­Izumida et al., 2013). Studies in early-lactating dairy cattle, which have extremely high energy requirements, have shown that energy requirements can be met by mobilizing fat stores from various sources within body (Humer et al., 2016; Contreras et al., 2017).

Following the conversion of muscle to meat, mitochondria and other metabolic mechanisms remain functional, and influence meat color development (Scheffler et al., 2015; ­Ramanathan et al., 2023). Dark-cutting beef has lower levels of glycolytic metabolites and greater levels of tricarboxylic acid cycle metabolites such as citric, malic, and fumaric acid (Ramanathan et al., 2020; Cônsolo et al., 2021; Kiyimba et al., 2024). Moreover, upregulation of metabolites required for mitochondrial function and amino acids in dark-cutting beef suggests muscle’s attempt to maintain homeostasis (Cônsolo et al., 2021; Antonelo et al., 2022). Furthermore, the upregulation of proteins associated with ATP production and mitochondria suggests that oxidative metabolism is predominant in dark-cutting beef (Gagaoua et al., 2021; Kiyimba et al., 2023). A study by Antonelo et al. (2022) reported that phosphatidylinositols were upregulated in dark-cutting beef than normal-pH beef from grass-fed *Bos indicus* bulls. In another study using early postmortem and 21-d-aged longissimus steaks from *Bos indicus* animals, changes in lipid associated with cell membrane and acylcarnitine were noticed in an intermediate pH category compared with normal and high pH groups (­Sartori et al., 2024).

Although much can be understood from metabolomics and proteomics, further research into the effects on the lipid profile of dark-cutting beef could help identify its biochemical basis. Furthermore, evaluating dark-cutting beef based on visual severity at the time of grading has been reported very little in the literature. The current study hypothesized that as dark-cutting severity increases, there will be subsequent changes in lipid profile. Therefore, the objective was to evaluate the impact of visual dark-cutting severity on the lipidome of unaged beef *longissimus lumborum*.

## Materials and Methods

Beef loins were purchased from a United States Department of Agriculture (USDA) Food Safety and Inspection Service inspected commercial plant. Therefore, institutional animal care and use committee approval was not requested for this study.

### Loin selection and procurement

Collection of beef strip loins (IMPS #180; *longissimus lumborum*) occurred at two midwestern commercial beef processors during January and April. At the time of grading by the USDA beef graders, carcasses (*n *= 6/treatment; 24 total loins) were classified based on the visual severity of dark-cutting condition. Treatments included a normal bright cherry-red (USDA Choice) control, shady (1/2 dark), moderate (2/3 dark), and moderately severe (full dark, not graded) dark-cutting beef. Carcasses selected were from A-maturity, grain-fed animals that were sourced from commercial feedlots, and were all of beef type, primarily black-hided cattle. Details of hot carcass weight, marbling score, fat thickness, and rib eye area are included in Table 1. Upon selection, carcasses were tagged, and identity was maintained through fabrication. Once fabricated, the loins were vacuum-packed and transported on ice to Oklahoma State University. At 48 to 60 h postmortem, two 2.54 cm steaks were fabricated from the anterior end of each loin. One steak was designated for lipidomic analysis, and the other steak was allocated for objective color.

**Table 1.** Mean carcass characteristics of different treatments

Treatment	Hot carcass weight (kg)	Marbling score	Rib eye area (cm^2^)	Fat thickness (cm)
**Normal**	938.5^a^	486^c^	15.0^a,b^	0.45^b^
**Shady**	764.6^c^	531^b^	13.5^c^	0.62^a^
**Moderate**	906.8^a,b^	562^b^	16.6^a^	0.48^a,b^
**Moderately severe**	879.8^b^	606^a^	14.1^b,c^	0.65^a^
**SD**	75.7	50.7	1.3	0.09

Marbling scores were converted to numeric values and ranged from 300 (Traces^00^) to 800 (Slightly Abundant^00^).

Fat thickness was measured at the 12^th^ rib.

^a,b,c^Means within a parameter lacking a common superscript differ (*P *< 0.05).

### pH

pH was taken at three random locations across the loin to measure ultimate pH before fabrication. Prior to use, the pH meter (HI99163; Hanna Instruments, Woonsocket, RI) was calibrated according to manufacturer instructions using pH 4 and 7 buffers. The three readings were averaged for each loin for statistical analysis.

### Objective color analysis

From the steak designated for color analysis, an approximately 5 cm × 5 cm freshly cut interior piece was allowed to bloom for 1 h at 4 ± 1.5 °C. Following the bloom period, surface color was read 3 times across the surface using a handheld spectrophotometer (HunterLab MiniScan 4500 L EZ; HunterLab Associates, Reston, VA). L*, a*, and b* values were recorded for each sample, and the three reads across the surface were averaged for each sample.

### Nontargeted lipidomics analysis

Samples from steaks designated for lipidomics analysis were trimmed without visible external and intramuscular fat and connective tissue. These separated steaks were minced, flash-frozen in liquid nitrogen, pulverized, packed into labeled 1.5 mL Eppendorf tubes, and stored at −80 °C until analysis. Lipidomic analysis was conducted at the University of California-Davis West Coast Metabolomics Center using the untargeted complex lipids assay. Briefly, samples were extracted according to the protocol established by Matyash et al. (2008). Samples were resuspended in 110 µL of a 9:1 methanol: toluene solution containing 50 ng/mL of 12-cyclohexylcarbamoyl amino dodecanoic acid (CUDA; a synthetic compound used for quality control) and shaken for 20 s. Following shaking, samples were sonicated for 5 min at room temperature and centrifuged at 16,100 g for 2 min. Samples were then aliquoted into 3 parts. For positive and negative mode lipidomics, 33 µL was aliquoted into a vial with a 50 µL glass insert. The remaining portion was placed into an Eppendorf tube to be used as a pool. After extracts were dried down and resuspended, the 4 ± 0.3 mg samples were injected onto the liquid chromatography mass spectrometry (LC-MS) system. Sample injections passed through the C18 column on the LC and then directly to the QTOF-MS/MS. Positive mode lipidomics were run on an Agilent 6546 with a scan range of m/z 120 to 1,200 Da with an acquisition speed of 2 spectra/s. In addition, 2 µL was injected onto an Acquity Premier BEH C18 1.7 µm, 2.1 × 50 mm column for the positive mode using a predetermined gradient. The additional aliquot was used to run negative mode lipidomics on the same liquid chromatography stack, mass spectrometer, and column as the positive mode. The acquisition rate was set at 2 spectra/s with a m/z 60 to 1,200 Da scan range. Mass resolution for Agilent 6,546 was set at 10,000 for ESI (+) and 30,000 for ESI (−). The details of the mass spectrometry settings are included in Supplementary File Table S1. Following acquisition, the data were normalized to the sum of the internal standards, and lipids were identified based on the Fiehn laboratory’s LipidBlast spectral library (Kind et al., 2013). A minority of internal standards were added during sample preparation to ascertain the quality of the extraction procedure, whereas the bulk of the internal standards were added during resuspension. The resuspension solution contained the Avanti UltimateSPLASH ONE lipidomics internal standards kit along with in-house internal standards. Nontargeted lipidomics is non-quantitative. The data provided are the peak heights of the extracted-ion chromatograms for each lipid.

### Statistical analysis

For pH, L*, a*, and b* data, simple statistics were generated using the Univariate procedure of SAS (Version 9.4; SAS Institute, Cary, NC). Analysis of variance was conducted using the Glimmix procedure with an alpha value of 0.05. The Kenward-Roger adjustment for denominator degrees of freedom was used in the analysis of variance. Mean separation occurred using Tukey’s *post-hoc* analysis and the LINES option of the LSMEANS statement to separate means when pairs were significantly different.

All statistical analysis of the lipidomic data was carried out using MetaboAnalyst (v. 6.0) and R Statistical Software (v. 4.2.0, R Core Team, 2023). For all analyses, samples were log_10_ transformed. A one-way analysis of variance was performed to determine if an overall difference existed between treatments, using a *P*-value and false discovery rate (FDR) cutoff of less than 0.05. Pairwise comparisons were conducted between two treatments using Student’s *t*-tests and an α value of 0.05 for the raw *P*-value. Partial least squares discriminant analysis (PLS-DA), variable importance in projection (VIP) plot, and heatmap analysis were also carried out using the functions available in MetaboAnalyst. The functional role of lipids was identified according to Han (2016). Enrichment analysis was conducted in MetaboAnalyst using the significantly different lipids, and the ggplot package in R was used to generate boxplots and volcano plots.

## Results

### pH and color evaluation

Moderately severe dark-cutting steaks had higher pH compared with other categories (normal < shady < moderate < moderately severe; *P *< 0.05; Table 2). Moderate and moderately severe dark-cutting loins had a lower (*P *< 0.05) L* and b* values than shady and normal loins (normal > shady > moderate = moderately severe). Moderate loins had a similar (*P *< 0.05) a* value to both moderately severe and shady loins, while normal-pH loins had the highest (*P *< 0.05) a* value than all other treatments.

**Table 2.** Summary statistics for pH, L*, a*, and b* values^1^ for the unaged normal, shady, moderate, and moderately severe dark-cutting strip loins^2^ (*longissimus lumborum*) utilized for lipidomics analysis

	Mean	Standard error	Minimum	Maximum
***pH***				
** Normal**	5.54^d^	0.11	5.15	5.69
** Shady**	5.96^c^	0.07	5.69	6.15
** Moderate**	6.38^b^	0.11	6.13	6.75
** Moderately severe**	6.55^a^	0.10	6.19	6.81
L* value				
** Normal**	38.21^a^	0.96	35.43	42.00
** Shady**	36.48^b^	1.61	32.59	42.21
** Moderate**	34.03^c^	0.87	31.59	37.44
** Moderately severe**	34.06^c^	1.25	29.55	36.41
a* value				
** Normal**	29.52^a^	0.95	27.08	33.26
** Shady**	25.07^b^	1.34	21.67	29.72
** Moderate**	23.25^b,c^	0.80	21.32	26.04
** Moderately severe**	21.94^c^	0.84	18.13	23.75
b* value				
** Normal**	20.89^a^	0.61	16.72	25.10
** Shady**	16.49^b^	0.81	12.25	22.28
** Moderate**	14.47^c^	0.67	10.09	20.48
** Moderately severe**	13.27^c^	0.58	7.22	16.64

1 L*, a*, and b* values generated from taking 3 reads across the surface of the interior surface of *longissimus lumborum* following a 1 h bloom in the presence of oxygen at 4 °C using a HunterLab MiniScan spectrophotometer (illuminant A, 10° standard observer angle, 2.54 cm aperture).

2 Dark-cutting loins were classified according to McKeith et al. (2016).

a,b,c,d Means within a parameter lacking a common superscript differ (*P *< 0.05).

### Lipidomic analysis

**Figure 1.**
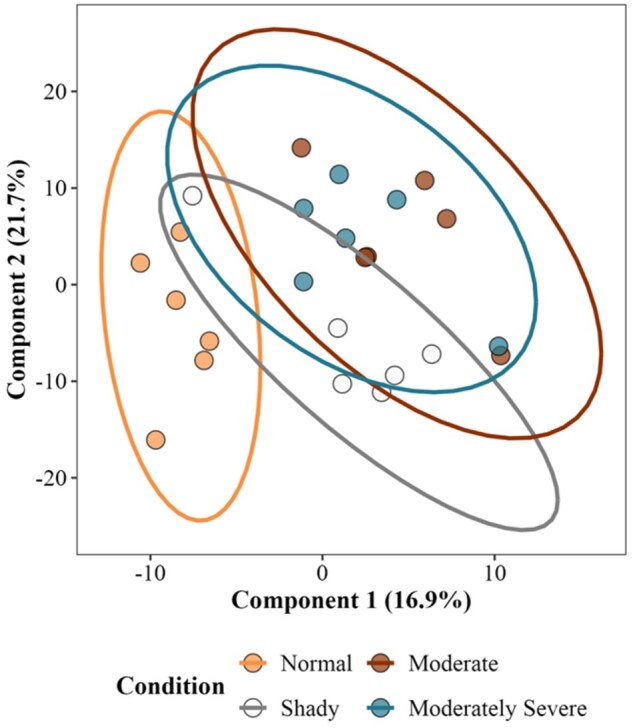
Partial least squares discriminant analysis (PLS-DA) of the lipid species present in unaged normal, shady, moderate, and moderately severe dark-cutting beef longissimus steaks.

**Figure 2.**
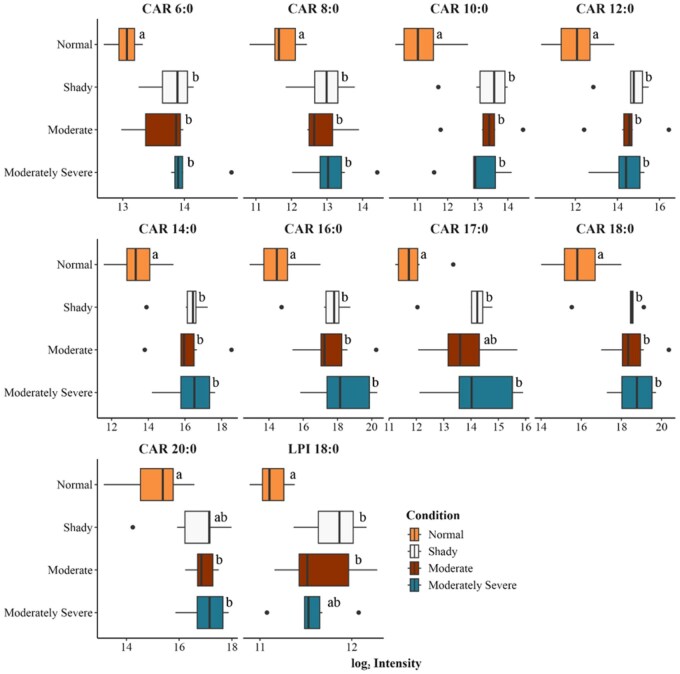
Boxplots of significantly different saturated lipid species present in unaged normal, bright cherry-red, shady, moderate, and moderately severe dark-cutting beef longissimus muscle. Different letters (a,b,c) in each treatment represent significant changes (*P* < 0.05). *n* = 6 replications for each treatment per condition.

**Figure 3.**
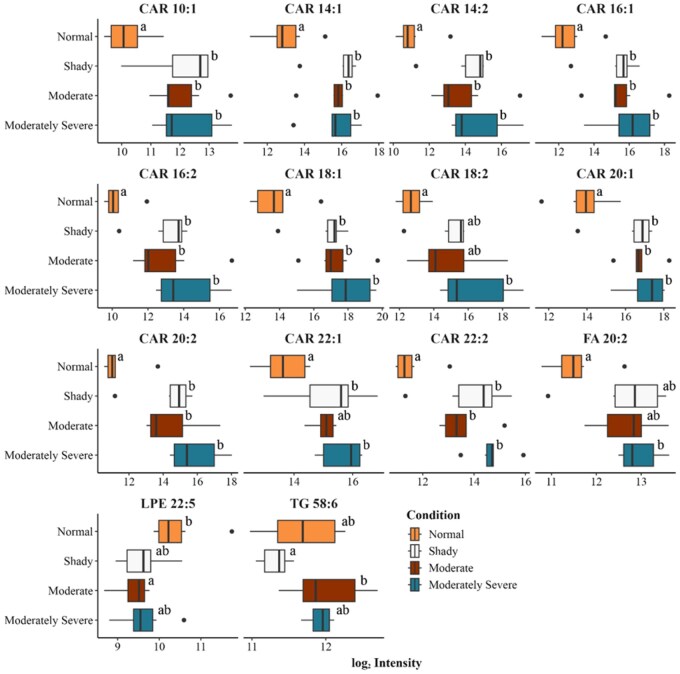
Boxplots of significantly different unsaturated lipid species present in unaged normal bright cherry-red, shady, moderate, and moderately severe dark-cutting beef longissimus muscle. CAR, acylcarnitine; FA, fatty acid; LPE, lysophosphatidylethanolamine; LPI, lysophosphatidylinositol; TG, triglyceride; Different letters (a and b) in each condition represent significant changes (*P* < 0.05). *n* = 6 replications for each treatment per condition.

A total of 379 lipid features were identified in the lipid library across four different treatments. Lipids belonging to various classes, such as triglycerides, sphingomyelin, phosphatidylserine, phosphatidylinositol, phosphatidylglycerol, ethanolamine glycerophospholipid, choline glycerophospholipid, mono- and diacylglycerols, acylcarnitine, ceramide, and free fatty acids, were determined in nontargeted lipidomics analysis. A PLS-DA was conducted to understand how identified lipids were separated using a multivariate supervised model. The PLS-DA plot indicates that there was separation between normal bright cherry-red beef and the moderate and moderately severe dark-cutting beef (**Figure 1**). There was overlap between the shady and normal treatments, as well as overlap between the moderate and moderately severe dark-cutting treatments. The analysis of variance identified 25 lipids as differentially abundant across the four treatments (**Figures 2 and 3**). Acylcarnitine species of varying carbon lengths were the predominant group of lipid species that differed (*P *< 0.05) between normal bright cherry-red and dark-cutting treatments. However, there were no differences among dark-cutting severities for most of the various carbon lengths of acylcarnitine. Lysophosphatidylethanolamine 22:5 was lower (*P *< 0.05) in moderate compared with normal bright red steaks. Sphingomyelin in shady dark-cutting was lower (*P *< 0.05) than in moderate and moderately severe.

**Figure 4.**
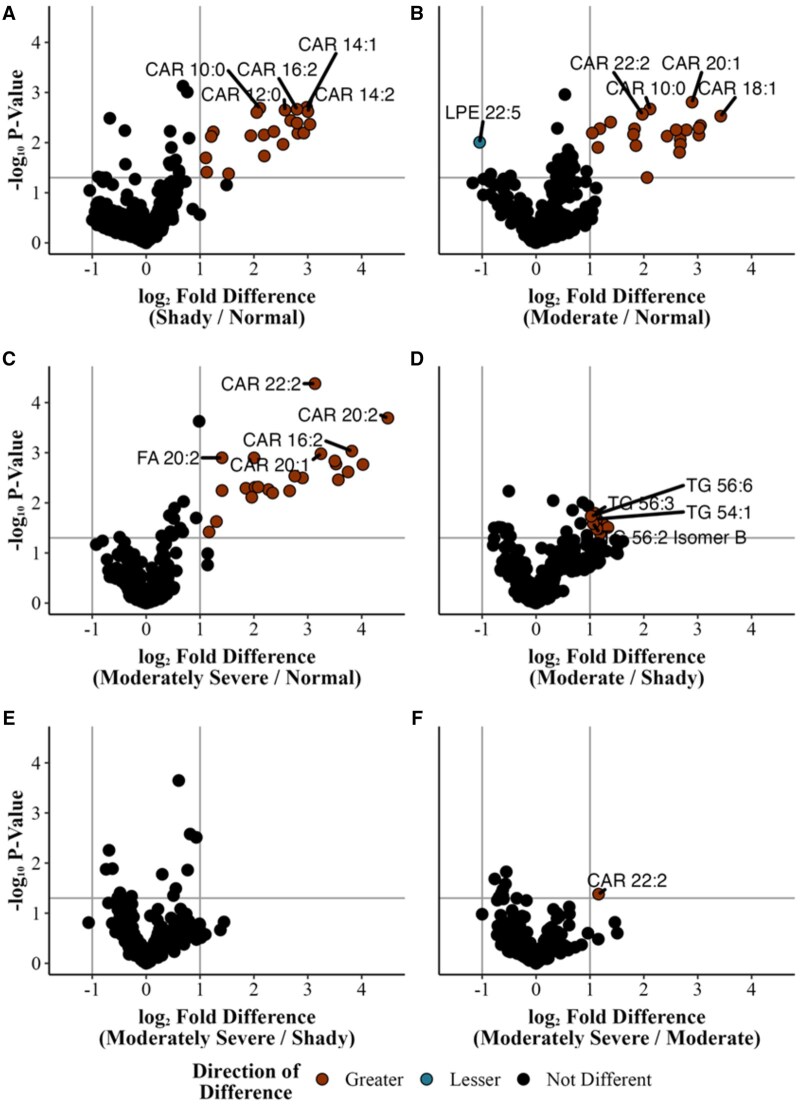
Volcano plots representing two-way comparisons of different treatments. Significantly different lipids (*P* < 0.05) are represented by brown or teal dots. *n* = 6 replications for each treatment per condition. Each plot contains pairwise comparisons of 379 identified lipids.

Pairwise comparisons were conducted to further understand how various treatments influenced lipid species (**Figure 4**). The comparison between moderately severe and normal indicated 23 lipid species were more abundant (*P *< 0.05), while the comparison between moderate and normal noted 21 lipids were more abundant, and one compound was less abundant. There was only one compound that differed (*P *< 0.05) between moderately severe and moderate, while no differences were noted when moderately severe and shady were compared. Comparisons between normal and shady dark-cutting beef identified 21 lipid species present at significantly higher relative abundance (*P *< 0.05), whereas comparisons between moderate and shady dark-cutting beef revealed 9 lipid species with significantly greater (*P *< 0.05) abundance.

**Figure 5.**
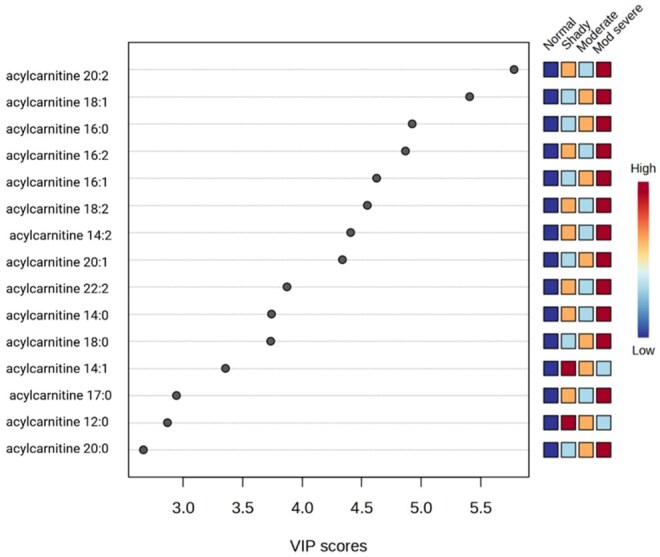
Features identified through partial least squares discriminant analysis (PLS-DA) that estimate the variable importance projection (VIP) of lipids from the current experiment. A variable’s importance within the model is indicated by a higher VIP score. *n* = 6 replications for each treatment per condition.

**Figure 6.**
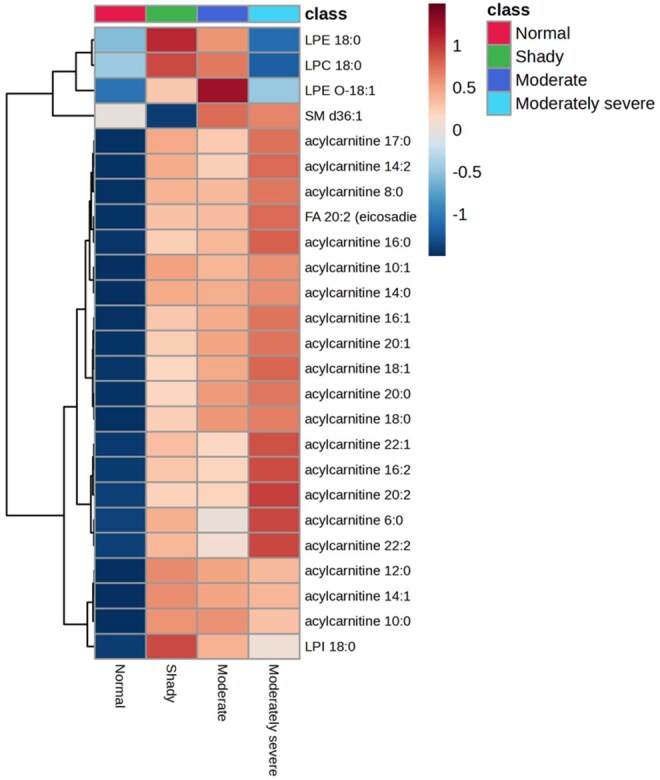
Heat map demonstrating the normalized intensity values of lipid species present in unaged normal bright cherry-red, shady, moderate, and moderately severe dark-cutting beef *longissimus lumborum* steaks. Greater abundance is indicated by red, while lower abundance is denoted by blue.

A VIP plot shows which metabolites or variables contribute the most to the PLS-DA model (**Figure 5**). It ranks the variables by importance, helping identify the key features that drive group separation. The VIP plot indicated that acylcarnitine 20:2 and acylcarnitine 18:1 were the top two lipid species that contributed to the separation of dark-cutting treatments. In general, medium and long-chain acylcarnitines contributed to the separation between normal and moderately severe. The heatmap clustered various lipid species into two major groups, especially acylcarnitines, between normal and various dark-cutting severities (**Figure 6**). A heatmap is a visual chart that uses colors to display the intensity or level of multiple variables simultaneously, helping to reveal patterns, clusters, and differences between groups.

**Figure 7.**
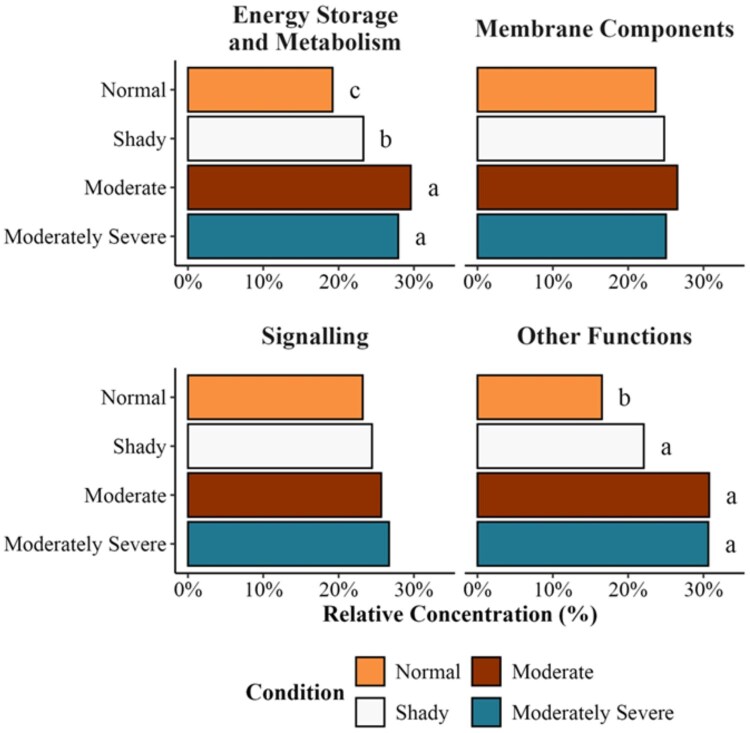
Relative concentrations of lipids grouped by the classification of lipid species based on their role in metabolism for the varying visual severities of dark-cutting beef. Letters (a-c) indicate significantly different (*P* < 0.05).

**Figure 8.**
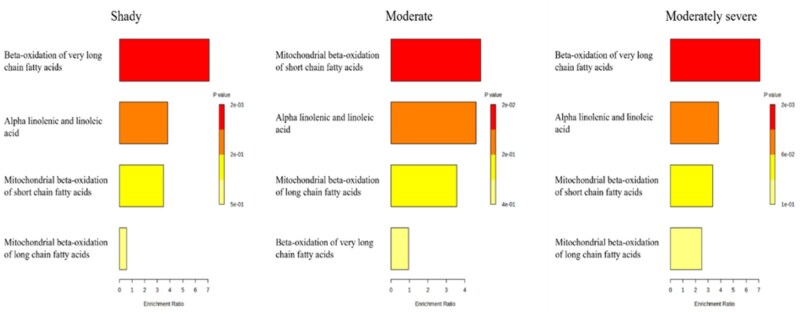
Enrichment analysis of significantly different lipid species in each dark-cutting severity when compared to normal beef. Enrichment analysis indicates that both shady and moderately severe dark-cutting beef showed greater differences (*P* < 0.05) in beta-oxidation of very long-chain fatty acids than other pathways. In contrast, moderate dark-cutting beef showed greater (*P* < 0.05) mitochondrial beta-oxidation of short-chain fatty acids (red color indicates greater significance and yellow color indicates less significance). Comparisons were made between each dark-cutting treatment and normal bright red treatment.

Lipid types related to energy storage had a greater (*P *< 0.05) relative concentration in moderate and moderately severe beef than in normal-pH beef (**Figure 7**). However, there were no differences between membrane components and signaling-related lipids. Pathway enrichment analysis performed in MetaboAnalyst, which evaluates whether predefined metabolic pathways are overrepresented based on the collective behavior of significantly different lipids. The current study indicated that shady and moderately severe dark-cutting beef exhibited greater enrichment (*P *< 0.05) of β-oxidation of very long-chain fatty acids relative to other metabolic pathways (**Figure 8**). In contrast, moderate dark-cutting beef showed greater enrichment (*P *< 0.05) of mitochondrial β-oxidation of short-chain fatty acids.

## Discussion

Metabolites are the final products formed from gene and protein synthesis. Hence, metabolite quantification helps to explain how these small molecules contribute to phenotype in biological systems. In the current study, there was an overabundance of numerous acylcarnitine lipid species of varying carbon length and saturation in the three dark-cutting severities. Acylcarnitine is primarily formed in the mitochondrial membrane (via carnitine palmitoyltransferase I) of various organs, such as liver, brain, skeletal muscle, and kidney, and within the mitochondrial matrix during fatty-acid β-oxidation (Burlina et al., 1989; Malaguarnera, 2012). Acylcarnitine is essential for the transport of long-chain fatty acids to mitochondria and peroxisomes, where it is converted into energy via β-oxidation and the production of acetyl-CoA (Stephens et al., 2007; Violante et al., 2013; Furuichi et al., 2014). Moreover, acylcarnitine also acts as a regulator and modulator in the selection of energy sources used by muscle, especially during periods of limited energy production (Stephens et al., 2007). Some studies suggest that glycogen storage in muscle cells may be partially regulated by acylcarnitine (Furuichi et al., 2014).

The proportion of short chain (C2-C5), medium chain (C6-C12), and long chain (C14-C22) acylcarnitine levels in tissue or plasma indicates the source of its formation (Makrecka-Kuka et al., 2017). For example, shorter chain acylcarnitine is formed from glucose, amino acids, and fatty acid degradation, while medium and long-chain acylcarnitine is formed mainly from fatty acid metabolism (Makrecka-Kuka et al., 2017). During high-intensity exercise in humans, acylcarnitine levels increased within 10 min and returned to normal levels during the rest period (Hiatt et al., 1989). In the current study, medium- and long-chain acylcarnitine levels were greater in dark-cutting severities, suggesting more fatty acid oxidation. In support, carnitine palmitoyltransferase 1 was overabundant in dark-cutting beef than in normal pH (Kiyimba et al., 2022). Relative concentrations of lipids related to energy storage were greater in moderate and moderately severe dark-cutting conditions, which supports a greater abundance of acylcarnitine. There were not many differences in lipid profiles among different shades of dark-cutting beef compared with normal bright red steaks. Metabolomic studies have reported a downregulation of glycolytic metabolites, but an upregulation of some of the tricarboxylic acid cycle metabolites in moderately severe dark-cutting beef (Ramanathan et al., 2020; Kiyimba et al., 2021). Harr et al. (2025) recently reported that 12, 21, and 43 metabolites related to glucose metabolism were significantly lower across shady, moderate, and moderately severe (pairwise comparisons to normal-pH beef) dark-cutting beef. Additionally, 11 metabolites were shared by shady, moderate, and moderately severe dark-cutting beef when compared to normal-pH beef (Harr et al., 2025). These suggest that amino acids, glycolytic, and tricarboxylic acid metabolites exhibit dark-cutting severity-dependent effects, whereas lipid species do not.

Only a limited number of studies have evaluated the lipidome of normal-pH or dark-cutting postmortem skeletal muscle. Recently, a multiple reaction monitoring profiling (mass spectrometric system without chromatographic separation) was employed to study the lipidome of high, intermediate, and normal pH beef *longissimus lumborum* from *Bos indicus*, and it was noted that acylcarnitine C12:0 and C14:0 were more abundant in intermediate pH than high pH and normal-pH beef (Sartori et al., 2024). In another research, 36 and 38 carbon phosphatidylinositol molecules were upregulated in high-pH beef than normal-pH beef from grass-fed *Bos indicus* animals (Antonelo et al., 2022).

Although lower levels of energy-providing metabolites may have contributed to the increased acylcarnitine levels observed across different shades of dark-cutting beef, the underlying mechanism remains unclear. However, studies with early lactation dairy cows, as well as human and mouse, can potentially provide further insights into our results. Dairy cows exhibiting high rates of lipolysis following calving have been shown to have higher concentrations of acylcarnitine species of varying carbon lengths than cows that had lower rates of lipolysis, suggesting that energy demands can in part be met by utilization of acylcarnitine-mediated fatty acid transport to produce ATP (Humer et al., 2016; Contreras et al., 2017). Humer et al. (2016) further speculated that mobilization of carnitine was stimulated by the need to transport fatty acids to mitochondria to generate metabolic energy, which is in line with the results of the current study. Dark-cutting beef has greater mitochondrial abundance compared to normal-pH steaks (McKeith et al., 2016; Ramanathan et al., 2020).

Besides the acylcarnitine species shared by all three dark-cutting severities, lysophosphatidylethanolamine 22:5 was lower in moderate compared with normal bright red steaks. Greater levels of lysophosphatidylethanolamine indicate membrane remodeling and oxidative stress in tissue (Makide et al., 2009). Sphingomyelin in shady dark-cutting severity was lower than in moderate and moderately severe treatments. Although sphingomyelin is integral to lipid metabolism, cell signaling, and membrane architecture (Li et al., 2024), its role in the current study is not clear.

### Limitations of the current study and future perspective

Research into understanding the biochemical basis of specific conditions is often challenging for various reasons. 1) When collecting product from a commercial facility, prior information about the live animal is frequently unknown or inaccessible, which poses a challenge to further interpreting the changes in acylcarnitine with specific biological conditions. 2) In selecting different severities of dark-cutting conditions, there exists significant variation in carcass traits, including marbling score and carcass weight, which can partially confound the differences in the dark-cutting conditions. Hence, future research warrants studying the lipidome of additional populations of dark-cutting beef, accounting for the above limitations, to understand the mechanisms underlying lipid mobilization for energy use. Additionally, combining lipidomics with metabolome and proteome data will provide further direction on the etiology of dark-cutting beef. Acylcarnitine has been used as a biomarker for several metabolic conditions because its concentrations reflect changes in fatty acid oxidation, mitochondrial function, and metabolic stress (Dambrova et al., 2022). For example, acylcarnitine levels are screened in newborns to study metabolic disorders. Hence, characterizing the role of acylcarnitine in dark-cutting conditions has the potential to predict its occurrence.

## Conclusion

The present study reported changes in the lipid profile across three dark-cutting severities. To the best of our knowledge, this is the first report of lipidomics research in grain-finished *Bos taurus* cattle exhibiting varying degrees of dark-cutting condition. Upregulation of acylcarnitine species of varying carbon lengths and saturation in all shades of dark-cutting beef suggests that lipid stores are being mobilized for energy metabolism via mitochondria. For example, acylcarnitine 20:2 and 18:1 explained most differences between dark-cutting severities and normal bright red steaks. Interestingly, there were no major changes in lipid profiles between moderate and moderately severe, or between moderately severe and shady, while the moderate and shady treatments showed nine lipids that differed. This research reconfirms alterations in energy homeostasis in dark-cutting conditions. In addition to differences in glycolytic, tricarboxylic acid, and amino acid levels, lipid profiles are also altered in dark-cutting conditions.

## Supplementary Material

skaf460_Supplementary_Data

## Data Availability

Data will be available on request.

## Abbreviations

ATP: adenosine triphosphate
ESI: electron spray ionization
IMPS: Institutional Meat Purchasing Specification
PLS-DA: partial least squares discriminant analysis
