# Supplementary material for: A preliminary nontargeted lipidomics analysis reveals greater acylcarnitine in dark-cutting beef longissimus lumborum across visual severity levels
Source: J Anim Sci. 2026 Jan 6;104:skaf460. doi: 10.1093/jas/skaf460 (PMC12884348; doi:10.1093/jas/skaf460)
Supplement: skaf460_Supplementary_Data [file skaf460_supplementary_data.docx]

**Supplemental File**

**A preliminary nontargeted lipidomics analysis reveals greater acylcarnitine in dark-cutting beef longissimus lumborum across visual severity levels**

Keayla M. Harr†, Madelyn A. Scott†, Eduardo Solano Pina dos Santos‡, Nara R. B. Cônsolo‡, Logan Johnson†, Gretchen G. Mafi†, Morgan M. Pfeiffer†, Ranjith Ramanathan†*

† Department of Animal and Food Science, Oklahoma State University, Stillwater, OK USA 74075

‡College of Animal Science and Food Engineering, University of São Paulo, Duque de Caxias Norte 225, Pirassununga/SP 13635-900, Brazil

Supplemental Table 1: LC–MS/MS running conditions, including column type, gradient, scan mode, mass/charge range, and other relevant acquisition parameters.

| Column | Waters Acquity Premier BEH C18 Column (1.7 µm, 2.1 mm x 50 mm) |
| --- | --- |
| Mobile phase A (+ mode) | 60:40 v/v acetonitrile:water + 10 mM ammonium formate + 0.1% formic acid |
| Mobile phase B (+ mode) | 90:10 v/v isopropanol:acetonitrile + 10 mM ammonium formate + 0.1% formic acid |
| Mobile phase A (- mode) | 60:40 v/v acetonitrile:water + 10 mM ammonium acetate |
| Mobile phase B (- mode) | 90:10 v/v isopropanol:acetonitrile + 10 mM ammonium acetate |
| Column temperature | 65°C |
| Gradient | 0 min 15% (B), 0–0.75 min 30% (B), 0.75-0.98 min 48% (B), 0.98-4.00 min 82% (B), 4.00-4.13 min 99% (B), 4.13-4.50 min 99% (B), 4.50-4.58 min 15% (B), 4.58-5.50 min 15% (B) |
| Flow rate | 0.8 mL/min |
| Injection volume | 2 µL (+); 5 µL (-) |
| Scan mode | Data-dependent acquisition |
| m/z range | 120-1200 Da (+); 60-1200 Da (-) |
